# Hematological profile of OSMF patients with increasing severity

**DOI:** 10.6026/973206300200353

**Published:** 2024-04-30

**Authors:** Vinod Sargaiyan, Simarjeev Singh, Ruchira Shukla, Abhishek Singh Tanwar, Tarang Mehta, Pranav V Manek, Bhumika J Patel, Keyur Jitendrakumar Patel

**Affiliations:** 1 Department of Oral Pathology and Microbiology, Maharana Pratap College of Dentistry and Research Center, Gwalior, M.P. India; 2 Department of Oral Medicine and Radiology, Gian Sagar Dental College and Hospital, Patiala, Punjab, India; 3 MDS, Private Consultant, Ortho expert Multispeciality Dental Clinic, Ambikapur, Chhattisgarh, India; 4 Department of Oral and Maxillofacial Surgery, Geetanjali Dental and Research Institute, Udaipur, Rajasthan, India; 5 Department of Oral and Maxillofacial Pathology and Oral Microbiology, Narsinbhai Patel Dental College and Hospital, Sankalchand Patel University, Gujarat, India; 6 Department of Oral Medicine and Radiology, Pacific Dental College and Research Centre, Udaipur, Rajasthan, India; 7 Department of Dentistry, Howard University, College of Dentistry, Washington DC, USA; 8 Government Medical College, Surat, India

**Keywords:** Haematological profile, OSMF, severity

## Abstract

Haematological profile of patients with oral sub mucous fibrosis (OSMF) and its correlation with the severity of OSMF is evaluated. The study comprised of sixty participants with clinical diagnoses. They were divided into smaller groups based on the OSMF stage. Sixty age and gender matched healthy controls were chosen among patients presenting for routine hematological examinations and free of systemic illnesses. Assessment of iron, hemoglobin, and red cell indices in all study participants was carried out. It was observed that the values of haematological tests like (Hb (g/dL), PCV, MCV (fl), MCH, MCHC, Iron (mg/dL) and Vitamin B12 (pg/Ml) was greater in normal subjects as compared to OSMF patients. Values were found to decrease further as the severity (staging) of OSMF increased among OSMF patients. The findings were statistically significant showing decrease in the values of different haematological parameters as the stage of OSMF progressed from stage I to stage III.

## Background:

Precancerous lesions, such as oral submucous fibrosis (OSMF), are clinically apparent abnormalities that are primarily noncancerous and frequently precede cancer of the oral cavity [1, 2]. Oral cancer can be diagnosed, its prognosis determined, and its progression tracked with the use of molecular markers that are present in bodily fluids like blood, saliva, and urine [3,4]. Tumor markers are chemicals that vary substantially in the serum as a tumor grows; these changes occur long before the disease is diagnosed [5,6].Even in cases of potentially malignant illnesses of the mouth, it is possible to predict whether a single individual with the fundamental biochemical deficiency would go on to acquire cancer in the future or not [7, 8]. Low hemoglobin (Hb) levels might affect the mucosa's consistency in the mouth. As biochemical markers, hemoglobin level - particularly serum iron level - is utilized to assess nutritional status. Iron, vitamin B-12, and folate deficiency will compromise the integrity of the oral mucosa [9, 10]. OSMF has been linked to hematological anomalies, including an elevated erythrocyte sedimentation rate (ESR), reduced serum iron, and increased iron-binding ability [10].Particularly among Asian populations, OSMF has become one of the most common potentially malignant illnesses (PMDs). Approximately 2.5 million people globally are impacted [11, 12]. Chewing areca nuts is the most frequently suggested etiological component for the illness; additional contributing variables include nutritional inadequacies, immunologic mechanisms, and a family history of the disease [13, 14]. Patients' clinical presentations vary based on the state of the illness at the time of being diagnosed, as the illness is progressive. Intolerant to hot foods, lip stiffness, tongue stiffness, and palate stiffness that reduces mouth opening, limited tongue motions, dysphagia, and diminished hearing in its advanced stages are among the most typical appearances [15, 16]. Sub-mucosal fibrosis, which affects the majority of the mouth, pharynx, and upper portion of the esophagus, is the disease's hallmark. It has been suggested that iron and vitamin deficits play a part in the genesis of OSMF.A great deal of research has been done to highlight their part in OSMF [17, 18]. A study found that compared to 200 participants in the normal control group, 104 OSMF patients had a considerably greater frequency of malnutrition. In malignancies including post cricoidal carcinoma along with esophageal tumors (Plummer-Vinson syndrome), iron has been investigated as a diagnostics and predictive marker. Cancer of the mouth and PMDs have been reported to have drastically changed serum iron levels [19, 20]. When a vitamin B12 deficit is corrected, medium to serious epithelial dysplasia can be reduced. For the oral mucosa to remain intact, both trace elements are necessary [21, 22]. The literature on the function of iron and vitamin B12 in OSMF is scarce. It is necessary to evaluate whether these trace components will change how OSMF develops and progresses [23, 24]. Further study in this area is needed to develop highly sensitive, precise, and quick assays for determining the severity of OSMF, given its multiple origins [25, 26].

Therefore, it is of interest to evaluate haematological profile of patients with OSMF and its correlation with the severity of OSMF.

## Materials and Method:

The study was cross-sectional. Patients with signs and symptoms of OSMF who visited the Department of Oral Medicine and Radiology for routine dental treatment made up the study population. The study comprised sixty participants with clinical diagnoses. They were divided into smaller groups based on the OSMF stage, as per the Thomas *et al.* categorization [19]. Sixty age and gender matched healthy controls were chosen from among patients presenting for routine hematological examinations and free of systemic illnesses.

Group 1: Normal subjects

Group 2: OSMF stage I

Group 3: OSMF stage II

Group 4: OSMF stage IV

## Criteria for inclusion:

[1] Patients clinically diagnosed with OSMF stage I to OSMF stage II

[2] Patients ready to participate in the study

## Criteria for exclusion:

[1] Individuals suffering from stage IV OSMF

[2] Clinically recognized oral mucosal lesions other than OSMF

[3] Previously managed OSMF cases

[4] Patients with lesions on their oral mucosa

[5] Individuals receiving medication for any other types of systemic illnesses

## Assessment of iron, hemoglobin, and red cell indices:

Five millilitres of fasting venous blood were drawn, and serum iron levels were estimated using the Ferrone technique and hemoglobin levels were calculated using Sahli's protocol. A differential pulse anodic stripping voltmeter (DPASV) and atomic absorption spectroscopy were used to examine the samples for trace elements (iron, copper) [12]. Vitamin B12 in human serum was quantitatively assessed using the chemiluminescent microparticle intrinsic factor assay [13].

## Statistical analysis:

The software utilized was IBM SPSS Statistics for Windows, version 20 (IBM Corp., Armonk, NY, USA). The standard deviations and mean values for each group were calculated using the Chi-square test. The normality of several parameters in the control and sample classes was assessed using the Kolmogorov-Smirnov test. When analyzing more than two means simultaneously, such as whether serum iron, vitamin B12, and hemoglobin levels significantly differed between the two groups, the independent t-test was employed. The study employed Karl Pearson's correlation coefficient methodology to examine the relationships between various parameters in both the control and sample groups.

## Results:

The mean age of study participants with different stages of OSMF was comparable with no statistically significant difference. The mean age was 35-40 years. Similarly the proportion of males was greater in each stage. However, the difference in proportion of males in different stages of OSMF was non-significant statistically (p>0.005) (Table 1).There were 60 normal subjects. 18 were having OSMF stage I, 22 were having OSMF stage II and 20 were having OSMF III (Table 2). It was observed that the values of haematological tests like (Hb (g/dL), PCV, MCV (fl), MCH, MCHC, Iron (mg/dL) and Vitamin B12 (pg/Ml) was greater in normal subjects as compared to OSMF patients. In OSMF patients values were found to decrease further as the severity (staging) of OSMF increased. The findings were statistically significant showing decrease in the values of different haematological parameters as the stage of OSMF progressed from stage I to stage III (Table 3).

## Discussion:

Several staging systems based on different aspects of OSMF have been suggested to aid classification [19]. Existing staging systems can be broadly categorized into clinical staging, histological staging, and clinicopathological staging. Compared to controls, OSMF patients in this study exhibited lower serum levels of iron, hemoglobin, and vitamin B12. Similar results were seen from other trials conducted globally [22, 23, 24]. This study was carried out to evaluate haematological profile of patients with OSMF and its correlation with the severity of OSMF. It was observed that the values of haematological tests like (Hb (g/dL), PCV, MCV (fl), MCH, MCHC, Iron (mg/dL) and Vitamin B12 (pg/Ml) was greater in normal subjects as compared to OSMF patients. In OSMF patients values were found to decrease further as the severity (staging) of OSMF increased. The findings were statistically significant showing decrease in the values of different haematological parameters as the stage of OSMF progressed from stage I to stage III. Ten (77 percent) of the Thirteen OSMF patients, according to a study, exhibited iron deficiency anemia (IDA). According to the current study, 93.6 percent had a much higher frequency of IDA than did the healthy control subjects [12-18]. Different outcomes have been found in several studies. According to a study, anemia was found in six percent of the 70 male OSMF participants and in eleven percent of the thirty-four female OSMF patients; however, there was no discernible difference in the prevalence of anemia between OSMF patients and the controls. Similar to what was seen in a study with 120 subjects, the current study likewise found a steady reduction in OSMF stage I to OSMF stage IV. The outcomes of our investigation align with other research findings [19-26].

The essential component of the human body, iron regulates several physiological as well as metabolic activities in addition to playing significant roles in RNA, DNA, antibody and collagen formation [6-12]. It is essential to the growth and upkeep of the oral mucosa. Clinical symptoms of iron deficiency anemia (IDA) include degeneration of the epithelium, weariness, achlorhydria, anxiety, dyspnea, and impaired memory. The condition known as dysphagia is caused by aberrant esophageal webs that are prone to malignant transformation. The three main histological characteristics of OSMF are enhanced collagen synthesis, thick corium, and epithelial atrophy [11-16]. The following explanations support the theory that OSMF has lower iron levels. To allow the epithelium to mature normally, cytochrome oxidase is needed. Epithelial atrophy brought on by IDA makes the mucosa susceptible to irritants. Because too much strongly cross-linked insoluble collagen Type I is produced, OSMF is essentially a disease of collagen metabolism [14- 18]. Both ascorbic acid and ferrous iron are necessary for the hydroxylation process of collagen. Serum iron levels are reduced as a result of using iron to hydroxyl-lateproline and lysine [15- 19].

OSMF was largely caused by IDA and was well treated with oral iron supplementation and antioxidants[22- 26].Iron-deficient humans and lab animals have been shown to exhibit chronic inflammation associated epithelial malfunction, two hallmarks of OSMF[12-17]. In several body organs, chronic inflammation is believed to be linked to an increased risk of cancer. Additionally, it has been observed that when head and neck carcinomas grow, serum ferritin levels rise and serum iron concentrations fall. For this reason, heme can be utilized as a follow-up measure for patients in addition to nutritional assessment [15- 20]. Participants with OSMF had serum vitamin B12 as well as folate concentrations within normal ranges, according to a study [24-26]. A preclinical Vitamin B complex shortage has been hypothesized in patients with OSMF with oral cavity vesiculation and ulcerations, as reported in a study [5-8]. In more severe cases, poor nutrition owing to reduced food intake may hasten the deficit, which could be the result rather than the etiology of the illness. Additionally, they stated that a lack of vitamins and iron combined with the host's malnourishment causes disruption in the lamina propria's inflammatory reconstructive response, which in turn causes inadequate healing along with scarification, all of which ultimately culminate in OSMF [9-12].The majority of the patients in this study tookgutkha, a concoction of tobacco and areca nut. Micronutrient depletion is one of the negative impacts of tobacco use. Low vitamin B12 levels could not be the only cause; they might also work in concert with environmental, genetic, and toxins to accelerate the malignant transformation process [14-22]. Because it aids in DNA synthesis and repairs damaged DNA, it is crucial for cancer prevention. While the inadequacies might not directly cause cancer, they do make people more vulnerable to the effects of other carcinogens [19, 20]. Because tobacco products along with areca nut use have increased in India, regardless of gender and among all age groups, the amount of cases of OSMF is notably high. Nevertheless, the significance of the hematological indicators is the subject of very few investigations [21-26].

## Conclusion:

The values of haematological tests were greater in normal subjects as compared to OSMF patients. Values were found to decrease further as the severity (staging) of OSMF increased among OSMF patients.

## Figures and Tables

**Table 1:** Demographic details of study participants

	**Age (Mean±SD) years**	**Male (%)**
Normal subjects	39.12± 1.21	78.23
OSMF stage I	36.14± 1.34	79.13
OSMF stage II	35.24± 1.15	80.14
OSMF stage III	38.15± 1.18	79.43
t value	2.13	3.14
P value	>0.05	>0.05

**Table 2:** distribution of study participants according to stage of OSMF

	**Number**
Normal subjects	60
OSMF stage I	18
OSMF stage II	22
OSMF stage III	20

**Table 3:** Comparison of values of different haematological tests in different stages of OSMF

	**Hb (g/dL)**	**PCV**	**MCV (fl)**	**MCH**	**MCHC**	**Iron (mg/dL)**	**Vitamin B12 (pg/Ml)**
Normal subjects	13.9±1.37	44.23±6.53	89.23±7.64	29.32±3.56	32.03±1.15	121.47±32.33	435.77±70.95
OSMF stage I	11.14±2.05	35.2 ±5.45	70.9±7.12	25.07±4.42	31.15±2.63	60.15± 12.37	221.22±44.88
OSMF stage II	10.03±1.12	32.3±1.37	67.1± 3.21	23.12±3.21	30.24±1.27	45.09±14.56	201.33±45.99
OSMF stage III	9.12±0.15	30.1±1.49	63.1±4.14	21.14±4.12	29.17±2.31	40.12±12.47	10.24±34.12
t value	7.6922	6.7233	9.1923	4.759	3.067	12.6255	12.4364
P value	<0.001	<0.001	<0.001	<0.001	<0.01	<0.001	<0.001
